# Bonding to Fluorosed Teeth: A Review of the Literature

**DOI:** 10.7759/cureus.56830

**Published:** 2024-03-24

**Authors:** Aram Alshehri, Faris Alrasheed, Khalid Alshayea, Talal Almubarak

**Affiliations:** 1 Restorative and Prosthetic Dental Sciences Department, College of Dentistry, King Saud Bin Abdulaziz University for Health Sciences, Riyadh, SAU; 2 College of Dentistry, King Saud Bin Abdulaziz University for Health Sciences, Riyadh, SAU

**Keywords:** adhesion, bonding, dentin, enamel, fluorosis

## Abstract

Excessive fluoride availability during tooth formation can cause structural alterations in enamel and dentin. These alterations may negatively impact the adhesion of various dental materials to teeth with dental fluorosis. The aim of this review of literature is to identify updates in bonding to fluorosed teeth and summarize relevant recommendations. Findings from the available literature suggest that bonding procedures may be carried out reliably on most fluorosed teeth with consideration to the severity of fluorosis and the employment of additional bond-enhancing measures for the severely involved teeth.

## Introduction and background

Fluorosis is a condition in which excessive fluorides are deposited into the hard and soft tissues of the body. Dental fluorosis results from high fluoride intake during tooth development, causing disturbances in tooth formation [1]. It is considered an endemic disease as it is more prevalent in certain communities where fluoride exposure is high. The global prevalence of dental fluorosis is about 32% [2]. Various sources of fluoride exposure are identified, including food, drinks, and dentifrices, with drinking water being the most common source of excessive exposure [2-4]. The incidence and severity of this developmental disorder are associated with the extent of fluoride exposure, including both the amount of fluoride and the length of the period of exposure [2,4,5].

Excessive fluoride availability during enamel formation hinders the removal of enamel proteins such as amelogenins, which results in sub-surface porosities and areas of hypomineralization, which can vary in appearance from mild white striations to brown stains or even pits in severely involved teeth [6-8]. Fluorosed dentin of permanent teeth was found to have increased interglobular dentin and accentuated incremental lines, according to von Ebner [9]. These structural alterations can negatively impact the adhesion of various dental materials to fluorosed teeth. Many studies have demonstrated a reduced bond strength of resin-based materials to teeth with fluorosis [10-16]. The aim of this paper is to review the available literature to identify updates in bonding to fluorosed teeth and summarize relevant recommendations.

## Review

Severity of fluorosis

The severity of dental fluorosis seems to influence the bond strength of different resin-based materials to fluorosed teeth. Significantly higher bond strength was found when bonding resin composite to enamel with mild fluorosis when compared to moderate or severe fluorosis [12,13,15]. The bond strength of the resin composite to dentin was also found to be negatively affected by fluorosis and inversely proportional to its severity [16]. The same was reported when testing the bond strength of glass ionomer-based restorative materials to fluorosed dentin [11]. Similarly, testing the bond strength of a self-adhesive resin cement and a resin-modified glass ionomer cement to fluorosed dentin demonstrated the same pattern of reducing bond strength with increased severity of fluorosis [14].

There are studies in the literature that could not demonstrate a reduction in bond strength in fluorosed teeth. One study found that fluorosis has no significant effect on the shear bond strength of resin composites with enamel [17]. Another study showed no difference in the tensile bond strength of resin composites to fluorosed and non-fluorosed enamel [18]. This may be explained by the fact that these studies, as well as many others, grind their enamel samples to achieve flat surfaces, which may be required for bond strength testing, removing the highly fluoridated surface enamel and resulting in an increased bond strength to fluorosed enamel [18,19]. This finding is further discussed in the following section.

Ground vs. unground tooth structure

While most bonding procedures are performed on prepared tooth structures, bonding to unground enamel may also be planned for the placement of orthodontic brackets, direct resin composite veneers or diastema closures, indirect no-preparation porcelain veneers, or even fissure sealants. Preparation of sound enamel does not seem to enhance the bond strength of the resin composite; however, a significantly higher bond strength of the resin composite to ground fluorosed enamel was found when compared to unground fluorosed enamel [18,19]. This indicates the beneficial effect of the removal of the hyper-mineralized, acid-resistant surface layer of enamel on the bond strength of resin composites to fluorosed teeth. This can be accomplished by conventional mechanical preparation using burs [18,19], through air-abrasion/sandblasting [20], or by micro-abrasion [21]. When the bond strength of porcelain veneers to prepared sound enamel and prepared moderately fluorosed enamel was compared, no difference was found due to the fact that the superficial hyper-mineralized enamel was removed by about 1 mm reduction, resulting in a similar bond strength to sound enamel [22].

Bonding systems

Selecting a bonding system among the various types available becomes more critical when the bond strength is thought to be compromised by dental fluorosis. Numerous bond strength testing studies investigate the performance of different bonding systems on fluorosed enamel and dentin.

Bond Strength to Enamel

The use of an etch-and-rinse adhesive system resulted in a significantly higher bond strength for fluorosed enamel compared to a two-step self-etching system. This finding was confirmed by all the studies that tested the bond strength of various bonding systems on fluorosed enamel [10,13,23,24]. When a two-step self-etching system was tested with and without the prior use of phosphoric acid etching on enamel, it was concluded that pre-etching with phosphoric acid significantly improved the bond strength of enamel with moderate and severe fluorosis [25]. The shear bond strength of a glass-ceramic (IPS Empress 2) to moderately fluorosed enamel was reported to be significantly higher when an etch-and-rinse luting system was used compared to a self-etching system [26].

Bond Strength to Dentin

The bond strengths of an etch-and-rinse system, a two-step self-etching system, and an all-in-one system to fluorosed dentin were compared, and it was found that the two-step system with a self-etching primer provided better adhesion to fluorosed dentin than the etch-and-rinse or all-in-one systems [16]. This was attributed to the chemical bond between calcium in hydroxyapatites and the functional monomer in the used self-etching primer, 10-methacryloyloxydecyl dihydrogen phosphate (10-MDP) [16]. While fluorosed enamel is more acid-resistant, fluorosed dentin seems to be more acid-susceptible, leading to superior bonding when a mildly acidic self-etching primer is used instead of phosphoric acid [16]. Another study tested the same 10-MDP-containing self-etching primer system on non-fluorosed and fluorosed dentin of various severities and found no difference in bond strength [27]. These studies suggest that the use of two-step self-etching adhesive systems may be beneficial for fluorosed dentin; however, when the bonding substrate is primarily enamel, etch-and-rinse systems are preferred.

Microleakage

Microleakage around class V resin composite restorations in fluorosed teeth with both enamel and dentin margins was examined, and it was found that the use of a two-step self-etching system resulted in poor marginal sealing on the enamel margin, which was greatly improved when selective etching with phosphoric acid was performed. Microleakage on the dentin margin was not influenced by additional etching with phosphoric acid [28]. Another study evaluated the sealing ability of etch-and-rinse, self-etch, and self-adhesive resin cements for luting porcelain veneers on enamel and dentin margins on fluorosed teeth. Their findings concluded that the best sealing on the enamel margin was provided by the etch-and-rinse resin cement and on dentin by the self-adhesive resin cement [29].

Bond enhancement

Various methods to enhance the bond strength of fluorosed teeth have been investigated in the literature. One logical method to think of is to increase the etching time of the highly fluoridated, acid-resistant fluorosed enamel. Longer enamel etching times with 37-40% phosphoric acid for teeth with fluorosis have been investigated for periods ranging from 30 to 150 seconds, resulting in a significant increase in the bond strength of resin composites to fluorosed enamel [17,30,31]. Doubling the etching time for 60 seconds on moderately fluorosed enamel produced etching patterns and bond strength comparable to sound enamel [30]. One study recommended increasing the enamel etching time with increased severity of fluorosis; therefore, etching enamel with mild fluorosis for 30 seconds just like non-fluorosed enamel, moderate fluorosis for at least 60 seconds, and severe fluorosis for at least 90 seconds to remove the hyper-mineralized surface layer; alternatively, this surface layer is ground away and then the sub-surface enamel is etched for 30 seconds [31]. Similarly, another study recommended extending the etching time of enamel with moderate fluorosis and sandblasting severely fluorosed enamel before acid etching [32]. Sandblasting or air-abrading enamel before acid etching is another method that can significantly increase the bond strength of orthodontic metallic brackets to fluorosed enamel [20]. Enamel micro-abrasion using a mixture of hydrochloric acid and silicon carbide particles (OpalustreTM) was found to significantly improve the bond strength of metallic orthodontic brackets to fluorosed enamel [21]. Moreover, as mentioned previously in this review, mechanical preparation using burs to remove the superficial hyper-mineralized enamel can significantly increase bond strength [18,19,22].

Other bond-enhancing measures have been suggested in the literature. Deproteinization of fluorosed enamel using 5.25% sodium hypochlorite (NaOCl) before phosphoric acid etching was found to eliminate organic matter, resulting in more effective etching that enhanced the bond strength of orthodontic brackets to fluorosed teeth [33]. The use of the Er:YAG laser (erbium-doped yttrium aluminum garnet laser) as a bond-enhancing measure for fluorosed teeth has been investigated with conflicting results that vary between no effect and increasing and decreasing the bond strength [34].

## Conclusions

Adhesive dentistry comprises a major part of today’s dental practice. Bonding various dental materials to teeth with fluorosis can pose a challenge. Methods to mitigate this problem have been investigated in the literature and are presented in this review. A limitation of the existing literature is that it mostly comes from laboratory studies; more clinical studies are needed to have a better understanding of the effect of fluorosis on bonding.

Bonding procedures may be carried out reliably on most fluorosed teeth with consideration to the severity of fluorosis and the employment of additional bond-enhancing measures for the severely involved teeth as needed. The severity of dental fluorosis seems to play a major role in determining the impact on bond strength; mild fluorosis does not seem to compromise the bond much, whereas more severely involved teeth may require additional bond strength-enhancing measures. Tooth preparation, which removes the superficial, highly fluoridated, acid-resistant tooth structure, appears to bring the bond strength back to normal. Most bonding issues are observed when bonding to non-prepared fluorosed teeth, such as in orthodontic bracket bonding, no-preparation resin composites, or porcelain veneers. For bonding to fluorosed enamel, etch-and-rinse adhesive systems are the best choice. Mildly fluorosed enamel can be etched at the same time as sound enamel; however, extending the etching time to at least double that of sound enamel is recommended for enamel with moderate and severe fluorosis, especially if not prepared. For bonding to fluorosed dentin, two-step self-etching adhesive systems showed superior results to other systems in laboratory studies. The use of a two-step self-etching adhesive system with selective phosphoric acid etching of enamel may be a suitable protocol for bonding to fluorosed teeth.

## References

[1] Møller IJ (1982). Fluorides and dental fluorosis. Int Dent J.

[2] Goodarzi F, Mahvi AH, Hosseini M (2016). The prevalence of dental fluorosis and exposure to fluoride in drinking water: A systematic review. J Dent Res Dent Clin Dent Prospects.

[3] Shahroom NS, Mani G, Ramakrishnan M (2019). Interventions in management of dental fluorosis, an endemic disease: A systematic review. J Family Med Prim Care.

[4] Di Giovanni T, Eliades T, Papageorgiou SN (2018). Interventions for dental fluorosis: A systematic review. J Esthet Restor Dent.

[5] Abanto Alvarez J, Rezende KM, Marocho SM, Alves FB, Celiberti P, Ciamponi AL (2009). Dental fluorosis: exposure, prevention and management. Med Oral Patol Oral Cir Bucal.

[6] Aoba T, Fejerskov O (2002). Dental fluorosis: chemistry and biology. Crit Rev Oral Biol Med.

[7] DenBesten PK, Heffernan LM (1989). Enamel proteases in secretory and maturation enamel of rats ingesting 0 and 100 PPM fluoride in drinking water. Adv Dent Res.

[8] DenBesten PK, Thariani H (1992). Biological mechanisms of fluorosis and level and timing of systemic exposure to fluoride with respect to fluorosis. J Dent Res.

[9] Fejerskov O, Yaeger JA, Thylstrup A (1979). Microradiography of the effect of acute and chronic administration of fluoride on human and rat dentine and enamel. Archives of oral biology.

[10] Ertuğrul F, Türkün M, Türkün LS, Toman M, Cal E (2009). Bond strength of different dentin bonding systems to fluorotic enamel. J Adhes Dent.

[11] Awliya WY, Akpata ES (1999). Effect of fluorosis on shear bond strength of glass ionomer-based restorative materials to dentin. J Prosthet Dent.

[12] Jaâfoura S, Kikly A, Sahtout S, Trabelsi M, Kammoun D (2020). Shear bond strength of three composite resins to fluorosed and sound dentine: in vitro study. Int J Dent.

[13] Liu S, Zhu Y, Gegen T (2020). Micromorphological analysis and bond strength comparison of two adhesives for different degrees of dental fluorosis. Applied Adhesion Science.

[14] Tan Y, Gu M, Li W, Guo L (2018). Effect of a filled adhesive as the desensitizer on bond strength of "Self-Adhesive Cements To" differently severity of fluorosed dentin. Microsc Res Tech.

[15] Torres-Gallegos I, A Martinez-Castañon G, Loyola-Rodriguez JP, Patiño-Marin N, Encinas A, Ruiz F, Anusavice K (2012). Effectiveness of bonding resin-based composite to healthy and fluorotic enamel using total-etch and two self-etch adhesive systems. Dent Mater J.

[16] Waidyasekera PG, Nikaido T, Weerasinghe DD, Tagami J (2007). Bonding of acid-etch and self-etch adhesives to human fluorosed dentine. J Dent.

[17] Ateyah N, Akpata E (2000). Factors affecting shear bond strength of composite resin to fluorosed human enamel. Oper Dent.

[18] Opinya GN, Pameijer CH (1986). Tensile bond strength of fluorosed Kenyan teeth using the acid etch technique. Int Dent J.

[19] Ermis RB, De Munck J, Cardoso MV (2007). Bonding to ground versus unground enamel in fluorosed teeth. Dent Mater.

[20] Suma S, Anita G, Chandra Shekar BR, Kallury A (2012). The effect of air abrasion on the retention of metallic brackets bonded to fluorosed enamel surface. Indian J Dent Res.

[21] Lupan I, Sachdev S, Sannoufi E (2011). Optimizing adhesion of orthodontic brackets to fluorosed teeth. Rom Jr Ora Reha.

[22] Ratnaweera PM, Fukagawa N, Tsubota Y, Fukushima S (2009). Microtensile bond strength of porcelain laminate veneers bonded to fluorosed teeth. J Prosthodont.

[23] Isci D, Sahin Saglam AM, Alkis H, Elekdag-Turk S, Turk T (2011). Effects of fluorosis on the shear bond strength of orthodontic brackets bonded with a self-etching primer. Eur J Orthod.

[24] Shida K, Kitasako Y, Burrow MF, Tagami J (2009). Micro-shear bond strengths and etching efficacy of a two-step self-etching adhesive system to fluorosed and non-fluorosed enamel. Eur J Oral Sci.

[25] Weerasinghe DS, Nikaido T, Wettasinghe KA, Abayakoon JB, Tagami J (2005). Micro-shear bond strength and morphological analysis of a self-etching primer adhesive system to fluorosed enamel. J Dent.

[26] Toman M, Cal E, Türkün M, Ertuğrul F (2008). Bond strength of glass-ceramics on the fluorosed enamel surfaces. J Dent.

[27] Ermiş RB, Gokay N (2003). Effect of fluorosis on dentine shear bond strength of a self-etching bonding system. J Oral Rehabil.

[28] Shafiei F, Abouheydari M (2015). Microleakage of class V methacrylate and silorane-based composites and nano-ionomer restorations in fluorosed teeth. J Dent (Shiraz).

[29] Shafiei F, Memarpour M, Jowkar Z (2017). Marginal sealing of a porcelain  laminate veneer luted with  three different resin cements  on fluorosed teeth. Int J Esthet Dent.

[30] Ng'ang'a PM, Ogaard B, Cruz R, Chindia ML, Aasrum E (1992). Tensile strength of orthodontic brackets bonded directly to fluorotic and nonfluorotic teeth: an in vitro comparative study. Am J Orthod Dentofacial Orthop.

[31] Al-Sugair MH, Akpata ES (1999). Effect of fluorosis on etching of human enamel. J Oral Rehabil.

[32] Silva-Benítez EL, Zavala-Alonso V, Martinez-Castanon GA, Loyola-Rodriguez JP, Patiño-Marin N, Ortega-Pedrajo I, García-Godoy F (2013). Shear bond strength evaluation of bonded molar tubes on fluorotic molars. Angle Orthod.

[33] Sharma R, Kumar D, Verma M (2017). Deproteinization of fluorosed enamel with sodium hypochlorite enhances the shear bond strength of orthodontic brackets: an in vitro study. Contemp Clin Dent.

[34] Zarif Najafi H, Moshkelgosha V, Khanchemehr A, Alizade A, Mokhtar A (2015). The effect of four surface treatment methods on the shear bond strength of metallic brackets to the fluorosed enamel. J Dent (Shiraz).

